# Advanced thermal metamaterial design for temperature control at the cloaked region

**DOI:** 10.1038/s41598-020-68481-6

**Published:** 2020-07-16

**Authors:** Muhammad Imran, Liangchi Zhang, Asit Kumar Gain

**Affiliations:** 1 Laboratory for Precision and Nano Processing Technologies, School of Mechanical and Manufacturing Engineering, University of New South Wales, Sydney, NSW 2052 Australia; 2 Department of Mechanics and Aerospace Engineering, Southern University of Science and Technology, Shenzhen, 518055 Guangdong China

**Keywords:** Mechanical engineering, Thermoelectric devices and materials

## Abstract

The present study focuses on maintaining the temperature magnitude around heat-sensitive components (cloaked region) in advanced electronic devices by introducing convective elements using extended surface fins. A finite element analysis confirmed that with the aid of the convection component to thermal cloaking, heat flux can be redirected around the cloaked region as well as control the temperature. The simulation results were verified by experiment under natural convection corresponding to the simulation assumptions. It was found that when the heat source maintains its temperature at 100 °C and the heat sink remains at 0 °C, the temperature within the cloaked region can reduce by up to 15 °C, from ~ 50 °C with conventional cloaking to 35 °C with a well-designed array of surface fins. It is worth noting that experimental results are consistent with the simulation results.

## Introduction

The advent of the transformation thermodynamics opened the avenues to mimic the concepts of transformation optics (TO) into thermal domain. The core of transformation thermodynamics is the characteristic of governing equations to be form invariant under coordinate transformation. This basic concept is applied to manipulate thermal flow, which made the thermal invisibility cloak possible. The mechanism of thermal cloaking involves redirecting the heat flow around the cloaked region due to transformed thermal conductivity as if nothing was placed in the cloaked region^1^. Thermal cloak possess two characteristics, (i) to produce zero temperature gradient region and (ii) to conceal anything placed within the cloaked region by turning the heat flow around the cloaked region. The key challenge of transformation thermodynamics is to achieve transformed material properties of being highly anisotropic and heterogeneous, which are severely different from natural materials. The concept of metamaterial, artificially structured to achieve desired material properties, provided the possibility to practically visualize the thermal cloak^2^ analogous to the cloaking in electromagnetics^3,4^. Thermal metamaterials initiated the possibility to practically realize several thermal functionalities such as thermal cloak^5–10^, thermal concentrator^11–14^, thermal rotator^15,16^, thermal illusion^17–20^, thermal camouflage^21–28^ etc. The recent research has been extended the use of thermal functionalities toward practical usage such as thermal encoding^29^, encrypted thermal printing^30^ etc.

Thermal cloak was first designed using thermal metamaterial made of concentric alternating rings of two isotropic homogenous materials to achieve the required anisotropy and heterogeneity of the bulk material^5^. Another approach of drilling varying sizes holes in a copper plate and filled it with polydimethylsiloxane (PDMS) to attain the desired thermal anisotropy was experimentally validated^6,7^. Furthermore, thermal cloaking was extended to three dimensional (3D) spherical cloak^31,32^. Thermal cloak has been designed theoretically in several shapes such as circular cloak^1,2,5,7,33,34^, rectangular cloak^25^, elliptical cloak^35^, diamond-shaped cloak^36^, sensu-shaped cloak^37^, arbitrarily-shaped cloak^38^ and so on^39^. However, the problem of keeping the temperature lower within the cloaked region persists. Although thermal cloaking can bend the heat flow around the cloaked region without disturbing the surrounding temperature distribution, temperature within the cloaked region keeps increasing over time^40^, which is undesirable for heat-sensitive components.

Additionally, the coordinate transformation technique is practically difficult to achieve perfect cloaking because of (i) the singularity in the theoretical model, (ii) the limited number of the applicable thermal conductivity materials, and (iii) incorporate only conduction heat transfer. Several investigations have thus been done on the manipulation of thermal convection along with conduction to improve the heat flow cloaking. For example, a convective element and moving fluid was integrated with a conductive system to achieve infinite thermal conductivity analogous to zero-index photonic metamaterial^41^. Mathematical framework with the help of coordinate transformation for thermal convection cloak was formulated using the thermal convection–diffusion equation, Navier-Strokes equation and Darcy’s law for steady^42^ and unsteady conditions^43^. Zhou et al. demonstrated a unified rotation as well as a cloaking transformation to enhance the heat manipulation functionalities^16^. Another approach for bending the heat flow direction with the help of architecture structure was introduced which can switch its functionalities based on the architecture structure orientation^44^. The current trends to control thermal flow are shifting towards phonon engineering to realize thermal functionalities at the microscale^45^. However, at this stage only heat conduction control is insufficient for keeping temperature lower at the cloaked region, which greatly limits this concept to be implemented in practical applications.

To solve the problem of elevated temperature within the cloaked region for an extended period of time, this study proposes the combined heat conduction–convection methods along with the coordinate transformation thermal cloak. An array of passive cooling mechanism, e.g., surface fins, is introduced to enhance the heat flow via convection and to maintain a lower temperature in the cloaked region. Finite element simulations and experiments are conducted to exploit the performance of the thermal metamaterial design. Possible optimal fin configurations are also investigated.

## Results

The temperature difference between two points drives the heat flux flow from higher temperature regions to lower temperature regions. Heat flow is divided into three modes: conduction, convection, and radiation. In solid metals, thermal conduction is more significant than convection and radiation. Heat conducts from higher temperature to lower temperature within a conductive domain.

A general equation of heat conduction without any internal heat-generation (Q = 0) can be written as,

1 $$ \nabla \cdot \left( {K\nabla T} \right) + \rho c{\raise0.7ex\hbox{${\partial T}$} \!\mathord{\left/ {\vphantom {{\partial T} {\partial t}}}\right.\kern-\nulldelimiterspace} \!\lower0.7ex\hbox{${\partial t}$}} = 0 $$

For steady-state case, ($$ {\raise0.7ex\hbox{${\partial T}$} \!\mathord{\left/ {\vphantom {{\partial T} {\partial t}}}\right.\kern-\nulldelimiterspace} \!\lower0.7ex\hbox{${\partial t}$}} = 0\user2{ })$$, Hence the simplified form of the Eq. (1), known as the Fourier equation of heat conduction, as;

2 $$ \nabla \cdot \left( {K\nabla T} \right) = 0 $$

**Figure 1:**
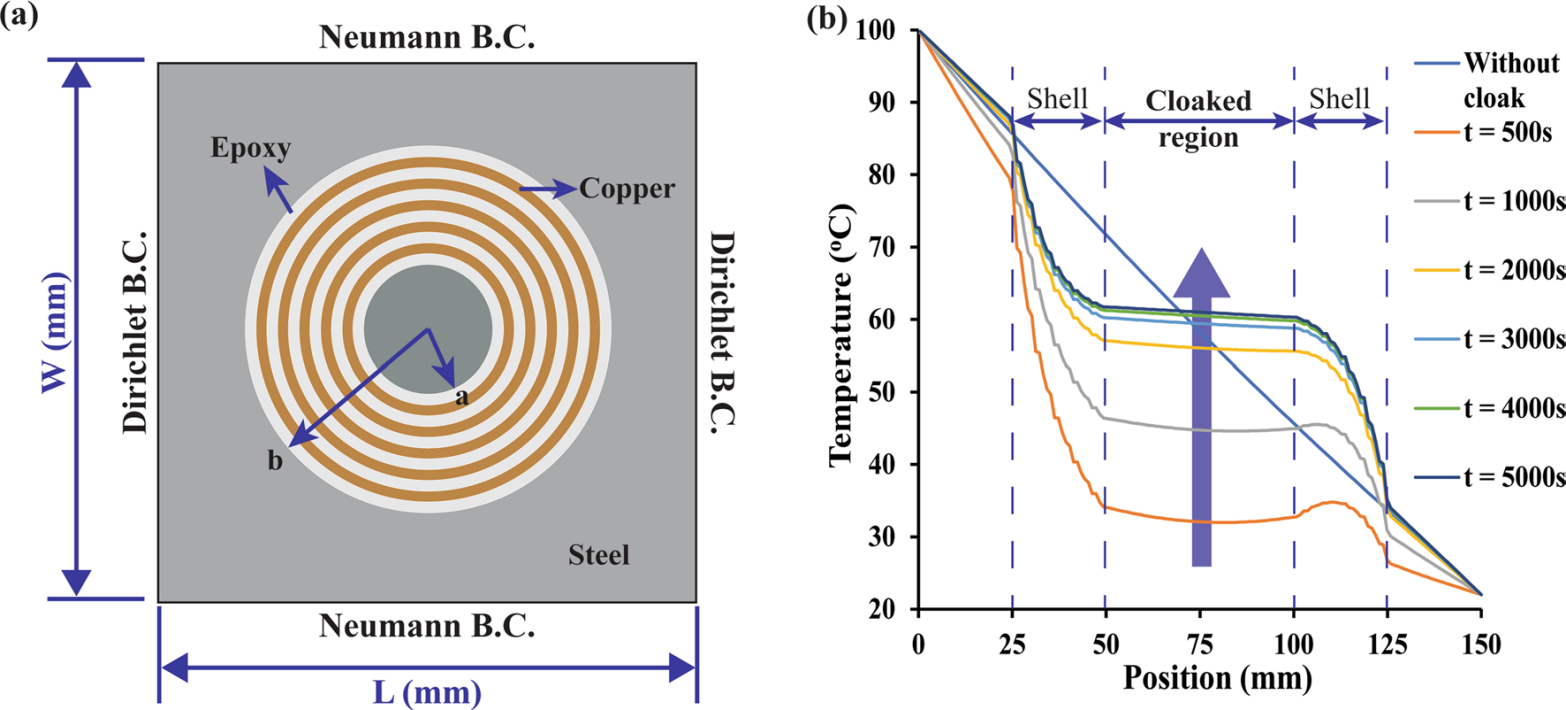
(**a**) Schematic of a conventional thermal cloak. Dirichlet boundary conditions are applied at the left side (T = 100 °C) and right side (T = 0 °C) and Neumann boundary conditions are applied at the top and bottom surfaces (∇*T*·*n* = 0). (**b**) Evolution of temperature with time obtained from FEM simulation of (**a**). (The graph results are obtained from Ansys-Thermal transient module version 19.2—https://www.ansys.com/ and compiled in Adobe illustrator version 17.0.0—https://www.adobe.com/au/products/illustrator.html).

Due to the form invariance characteristic of the Fourier equation of heat conduction, it keeps its form after transformation^7,46^.

3 $$ \nabla^{\prime} \cdot \left( {K^{\prime}\nabla T^{\prime}} \right) + \rho^{\prime}c^{\prime}{\raise0.7ex\hbox{${\partial T^{\prime}}$} \!\mathord{\left/ {\vphantom {{\partial T^{\prime}} {\partial t}}}\right.\kern-\nulldelimiterspace} \!\lower0.7ex\hbox{${\partial t}$}} = 0 $$

where,

4 $$ k^{\prime} = \frac{{A \cdot k \cdot A^{t} }}{\det \left( A \right)} $$

and

5 $$ \rho^{\prime}c^{\prime} = \frac{\rho c}{{det\; \left( A \right)}} $$

where “*A*” is the Jacobian transformation and “*A*^*t*^” is the transpose of matrix “*A*”. Cloaking is designed by linear transformation of coordinates from a circular region of radius “*b*” (r ≤ b) to the annular region of the inner radius as “*a*”. (a ≤ r ≤ b), as shown in Fig. 1a. The mathematical correspondence for cylindrical cloak and spherical cloak is as follows;

$$ {\text{Cylindrical }}\;{\text{cloak}}:\;r^{\prime} = b + \frac{{r\left( {b - a} \right)}}{b}, \;\;\;\theta^{\prime} = \theta ,\;\;\,z^{\prime} = z $$

$$ {\text{Spherical}}\;{\text{ cloak}}:\;r^{\prime} = b + \frac{{r\left( {b - a} \right)}}{b},\;\;\;\theta^{\prime} = \theta , \;\,\;\varphi^{\prime} = \varphi $$

The Jacobian matrix can be calculated as;

$$ A = \frac{{\partial \left( {x^{\prime},y^{\prime},z^{\prime}} \right)}}{{\partial \left( {x,y,z} \right)}} = \frac{{\partial \left( {x^{\prime},y^{\prime},z^{\prime}} \right)}}{{\partial \left( {r^{\prime},\theta^{\prime},z^{\prime}} \right)}} \cdot \frac{{\partial \left( {r^{\prime},\theta^{\prime},z^{\prime}} \right)}}{{\partial \left( {r,\theta ,z} \right)}} \cdot \frac{{\partial \left( {r,\theta ,z} \right)}}{{\partial \left( {x,y,z} \right)}} $$

From these equations, the anisotropic conductivity of the transformed thermal cloak is obtained as,

6 $$ \frac{{k^{\prime}}}{k} = \frac{b}{b - a}diag\left( {\begin{array}{*{20}c} {\left( {\frac{b - a}{b} \frac{r}{{r^{\prime}}}} \right)^{2} } & 1 & 1 \\ \end{array} } \right) $$

The governing equation of convection heat transfer, Q_conv_, is as follows,

7 $$ Q_{conv} = hA\left( {T_{w} - T_{\infty } } \right) $$

where, h = convection heat transfer coefficient, A = effective surface area, T_w_ = fin wall temperature, and T_∞_ = environment temperature/room temperature.

The fin effectiveness can be calculated by;

8 $$ \epsilon_{fin} = \frac{Heat\, transfer\; without \,fins}{{Heat\; transfer \;with \,fins}} = \frac{{Q_{conv} }}{{Q_{fins} }} $$

The fin effectiveness can be enhanced by; (i) using high thermal conductivity material e.g., aluminium, copper, (ii) higher ratio of surface area to the perimeter of the fins, (iii) thin and closely placed fins for natural convection rather than thick fins, and (iv) smooth airflow path within the fins.

### Finite element simulation of proposed design

Numerical simulations of the conventional thermal cloak were conducted to understand the cloaking phenomenon using the finite-element method (FEM) based on commercially available software Ansys v19.2 in the heat transfer system. A rectangular-shaped base plate (210 × 100 mm size) with a central hole of radius 52.5 mm and circular-shaped cloaked region of radius 25 mm was considered. Around the cloaked region, thermal metamaterial composed of concentric layers of copper and epoxy resin was fitted which joined the base plate and cloaked region. Dirichlet boundary conditions were applied at the side surfaces i.e. left side: T_H_ = 100 °C and right side: T_L_ = 0 °C and Neumann boundary conditions were applied at the top and bottom surface i.e. $\nabla T \cdot n = 0$. The thickness of all the components was 1 mm. The simulation setup presented in Fig. 1a.

**Figure 2:**
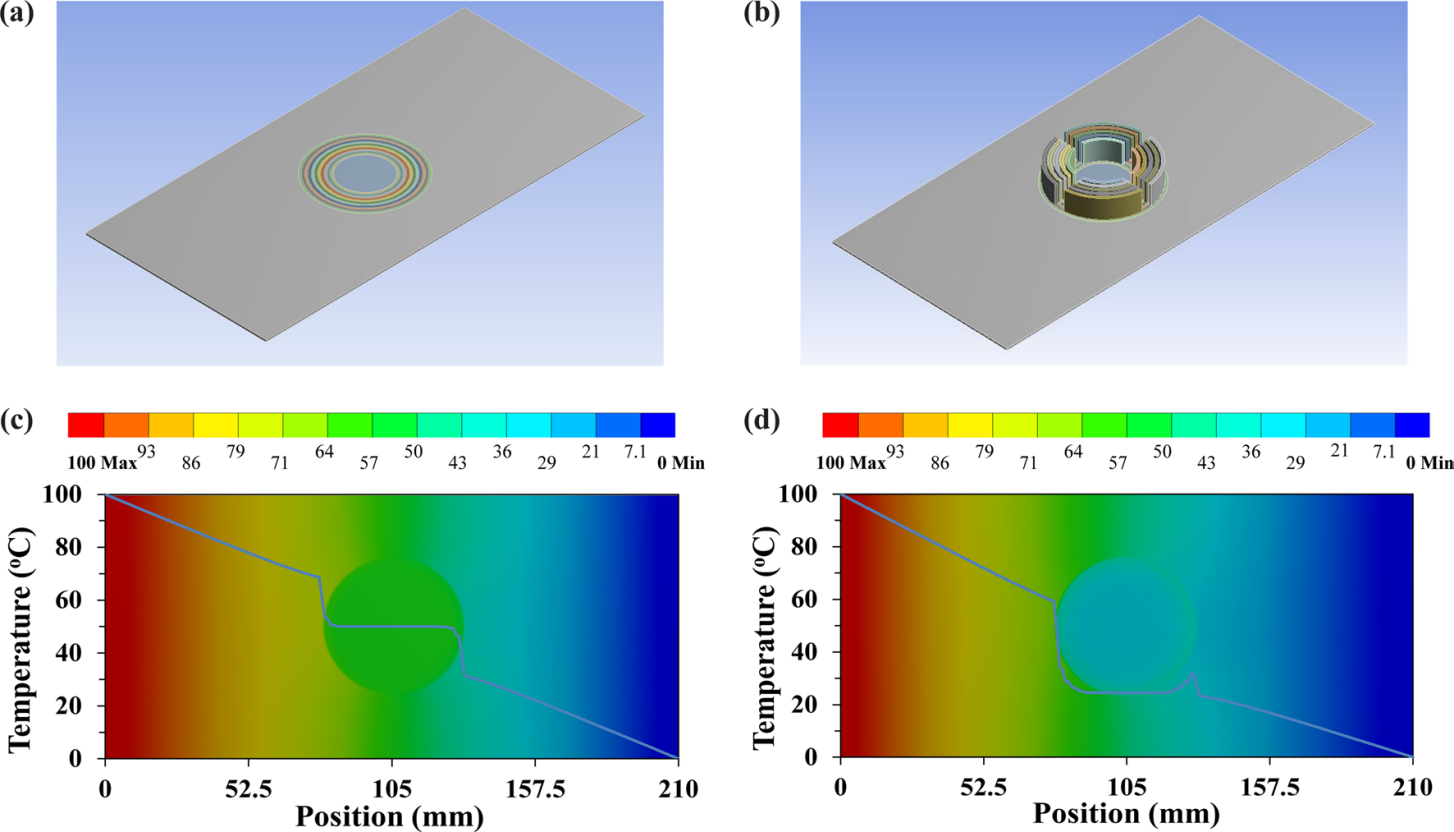
Finite element simulation performance comparison of conventional cloak and proposed convective element cloak. (**a**) Conventional cloak. (**b**) Proposed convective element cloak design. (**c**) Temperature gradient and cloaked region temperature for a conventional cloak. (**d**) Temperature gradient and cloaked region temperature for a convective element cloak. (The results are obtained from Ansys-Thermal transient module version 19.2—https://www.ansys.com/ and compiled in Adobe illustrator version 17.0.0—https://www.adobe.com/au/products/illustrator.html).

The FEM simulation shows an accurate thermal cloak by having a constant temperature in the cloaked region presented in Fig. 1b. However, when this case was run for several hours, the magnitude of temperature within the cloaked region kept rising even though the temperature gradient was zero. These theoretical results confirmed that the thermal cloaking mechanism was not suitable for modern electronic devices as these are being used for longer period continuously. The convectional thermal cloaking only redirects the heat flow without reducing the temperature at the cloaked region. To overcome this problem and reduce the temperature within the cloaked region, a combined heat conduction–convection heat transfer mechanism is proposed. To do this, convective elements, surface fins were attached with the thermal metamaterial as shown in Fig. 2a. The fins helped to reduce the temperature while keeping the cloaked region invisible to heat flow. The circular-shaped fins were attached with the copper rings, due to its high thermal conductivity, to have passive convection as shown in Fig. 2c. The results showed a drastic reduction in temperature at the cloaked region from ~ 50 to ~ 26 °C as shown in Fig. 2b, d.

**Figure 3:**
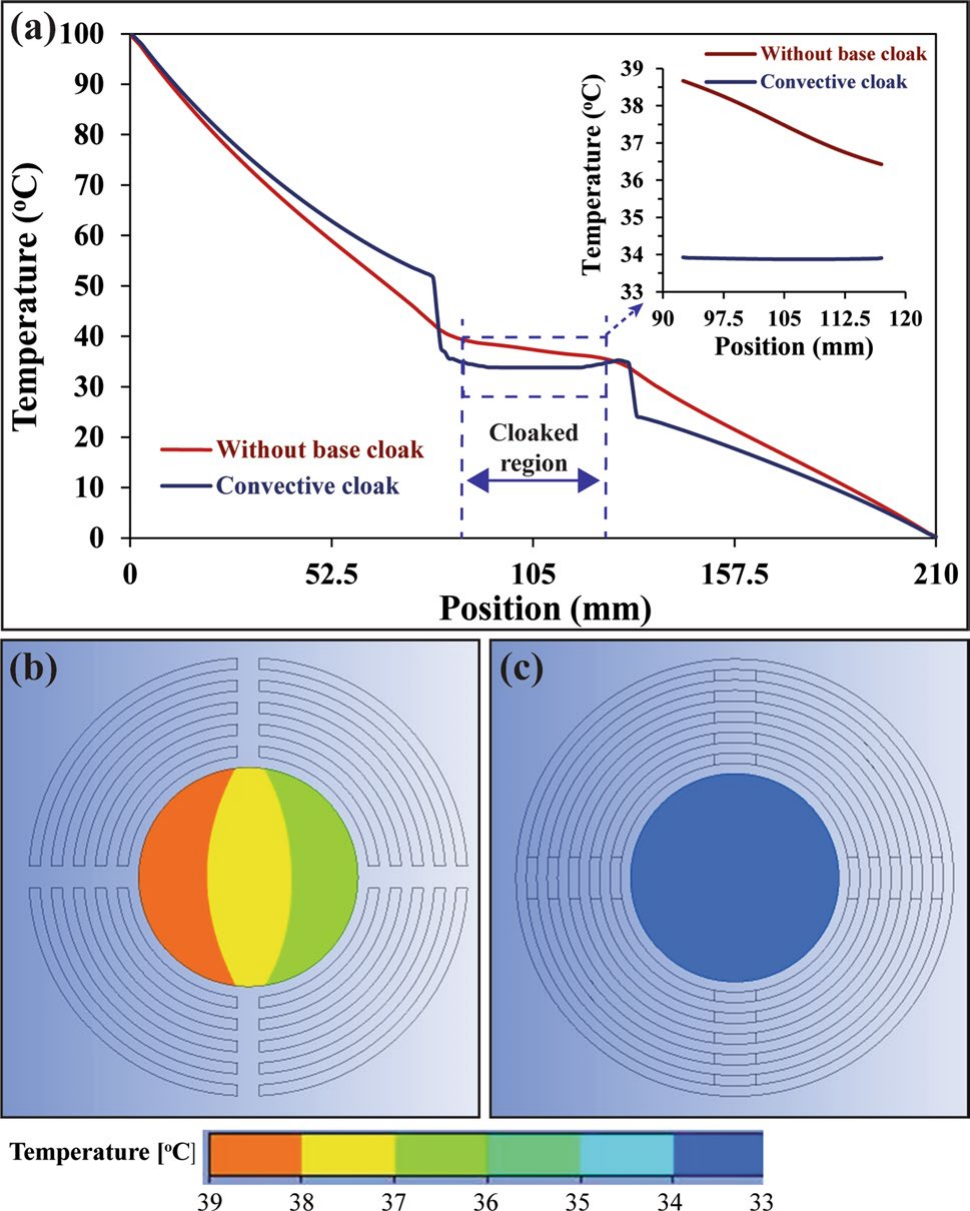
Finite element analysis of base cloak effectiveness. (**a**) Comparison of temperature distribution for proposed convective element cloaking without and with base cloak. Temperature contour (temperature gradient) at the cloaked region for proposed convective element cloaking (**b**) without base cloak (**c**) with base cloak. (The results are obtained from Ansys-Fluent module version 19.2—https://www.ansys.com/ and compiled in Adobe illustrator version 17.0.0—https://www.adobe.com/au/products/illustrator.html).

To understand the effectiveness of the base cloak on the proposed design, a finite-element simulation was conducted and results were presented in Fig. 3. According to this simulation results, it was confirmed that the addition of convective (passive cooling) elements significantly reduced the temperature at the cloaked region even without base cloak as compared with conventional cloak presented in Fig. 2c. However, there is considerable high temperature gradient [shown in Fig. 3b], compared with the convective element cloaking with the base cloak [shown in Fig. 3c], which is not desirable. Hence, convective element with base cloak was considered for experimental analysis which are presented in the following section.

### Experimental realization of convective element cloaking performance

**Figure 4:**
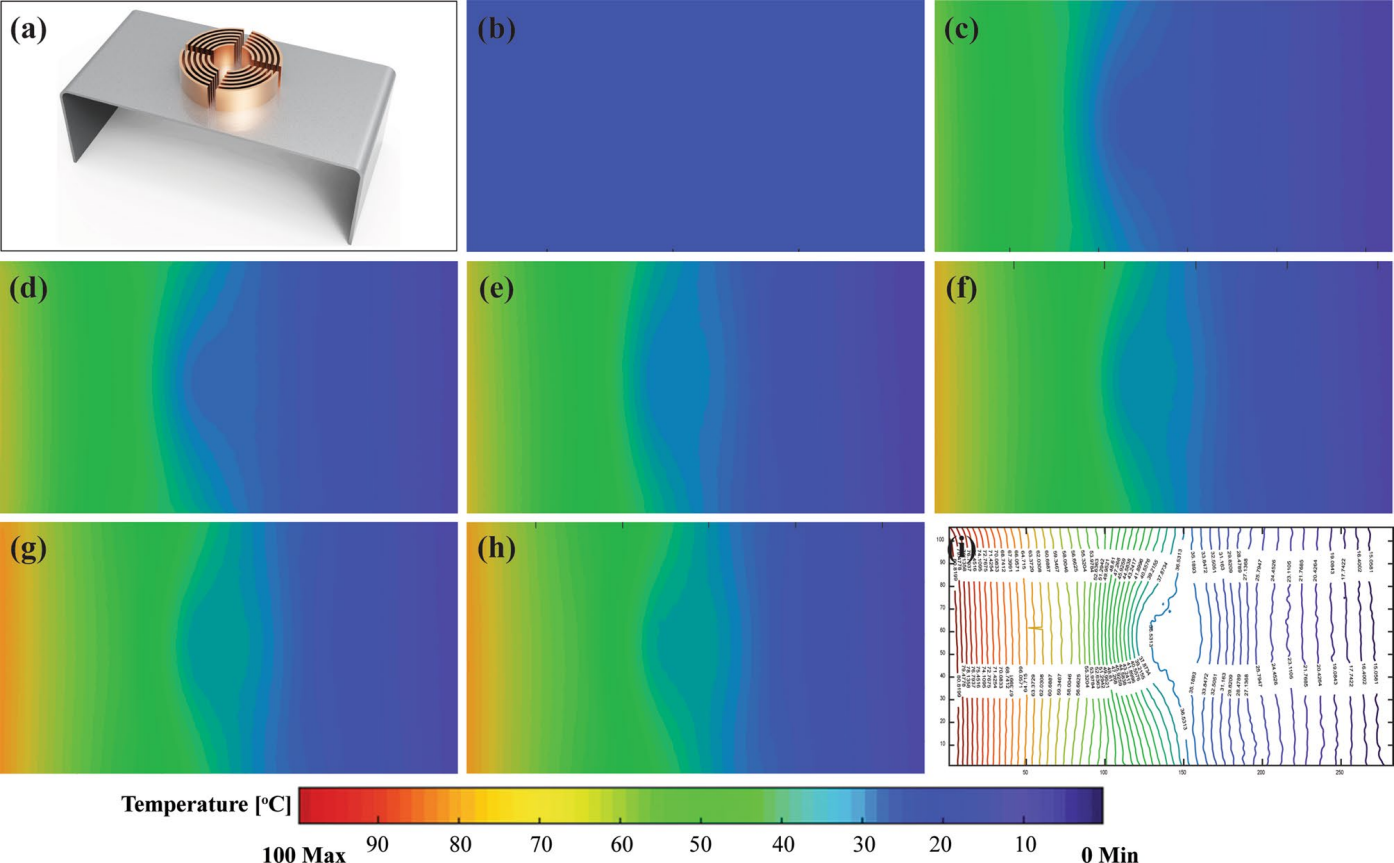
Experiment results recorded by infrared camera FLIR E40. Temperature distribution at interval to time “t” for (**a**) Convective element with grooved fins specimen (**b**) t = 0 min (**c**) t = 5 min (**d**) t = 10 min (**e**) t = 30 min (**f**) t = 45 min (**g**) t = 75 min (**h**) t = 90 min (**i**) t = 480 min. The contour plot of temperature distribution at the end of the experiment. (The results are obtained from FLIR tool version 5.13.18031.2002—https://www.flir.com.au/products/flir-tools/ and compiled in Adobe illustrator version 17.0.0—https://www.adobe.com/au/products/illustrator.html).

To examine this result experimentally, specimens with identical geometric parameters were fabricated. The experimental conditions were kept analogous to the simulation shown in Fig. 1a. Figure 4 illustrates the heat signatures changing transiently on the convective element cloaking from the initial state at t = 0 min until it’s reached steady-state at approximately t = 90 min from Fig. 4b–h, respectively. In Fig. 4i, the temperature contour lines were presented to quantify the temperature at any point on the specimen at t = 480 min. The uniform temperature contour at the centre in Fig. 4 corresponds to the cloaked region with perfect cloaking. However, the temperature recorded at the cloaked region was approximately 34.5 °C. The experimental results exhibited a higher temperature compared to the FEM simulation results presented in Fig. 2b. The main reason for seeming higher temperature in experiments was due to the varying convection coefficient during the transient state in the experiment. While the constant convection coefficient was considered by the FEM thermal module simulation throughout. For further analyses, Ansys thermal module was not considered for convection heat transfer analysis. Hence, later Ansys Fluent (CFD) v19.2 was used for numerical simulations. The full-scale design model was built in the fluent pre-processor. Since a passive temperature control mechanism was used, the natural convection heat transfer model was considered along with the effect of gravity. The air domain was designed substantially large, with a dimension of 600 × 600 × 400 mm, so that free stream temperature remained constant (about 23 °C) throughout the experiment. The working fluid e.g., air was considered as incompressible throughout the analysis. For the coefficient of convection heat transfer, the interaction of fins and air (solid–fluid coupling) was defined instead of assigning any constant value to mimic the experimental analysis. Shared topology was applied for contact in-between the components such as baseplate, concentric rings, cloaked region and fins. Heat source temperature (100 °C) was assigned at the one end and heat sink temperature (0 °C) on the other end of the baseplate. The room temperature (23 °C) was considered constant throughout the experiment. The time span for the simulation was considered as 8 h (480 min).

**Figure 5:**
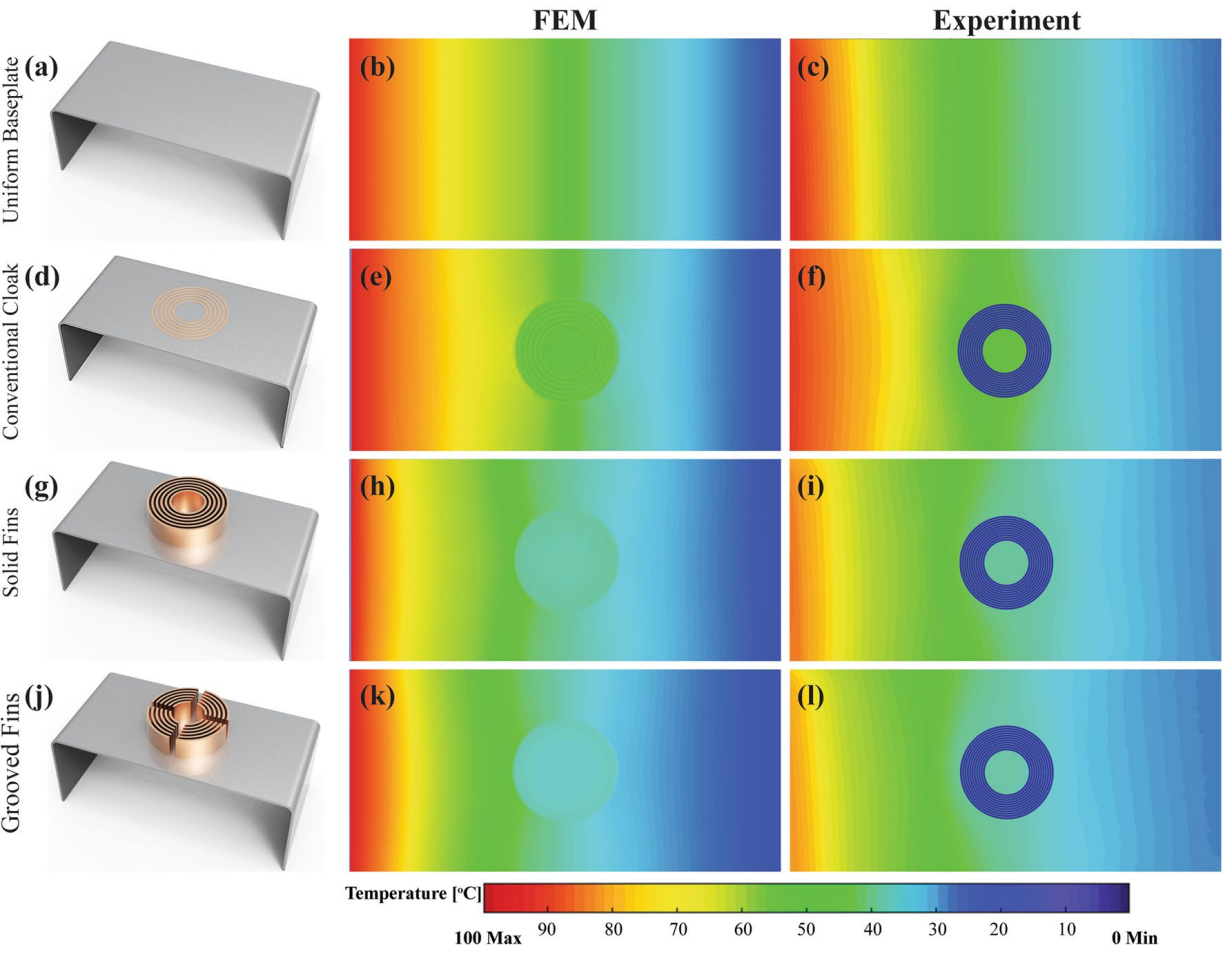
Comparison of experimental and Finite element (Fluent) results (**b**, **e**, **h**, **k**) FEM simulation and (**c**, **f**, **i**, **l**) Experimental results, at t = 480 min, for (**a**) Uniform baseplate without cloak, (**d**) Conventional cloak, (**g**) Convective element cloak with solid fins and (**j**) Convective element cloak with grooves. (The simulation results are obtained from Ansys-Fluent module version 19.2—https://www.ansys.com/, the experimental results are obtained from FLIR tool version 5.13.18031.2002—https://www.flir.com.au/products/flir-tools/ and compiled in Adobe illustrator version 17.0.0—https://www.adobe.com/au/products/illustrator.html).

The comparison of fluent simulations and experimental results for four specimens are presented in Fig. 5. The considered specimens are named by (i) uniform baseplate, (ii) conventional cloaking, (iii) convective element cloaking with solid fins and (iv) convective element cloaking with grooved fins as illustrated in Fig. 5a, d, g j, respectively. Both simulation and experimental results are presented in Fig. 5 after running up to 480 min (8 h). As illustrated in Fig. 5, the isothermal lines were smoothly distributed on the baseplate with slight distortion in experimental results. Uniform temperature distribution observed within the cloaked region without any temperature gradient in all cases. This indicated a perfect cloaking behavior as shown in Fig. 5d–l. On the other hand, owing to the addition of convective element on conventional cloaking, the uniform temperature value within the cloaked region reduced drastically (Fig. 5g–l), in comparison with conventional cloaking (Fig. 5d–f).

**Figure 6:**
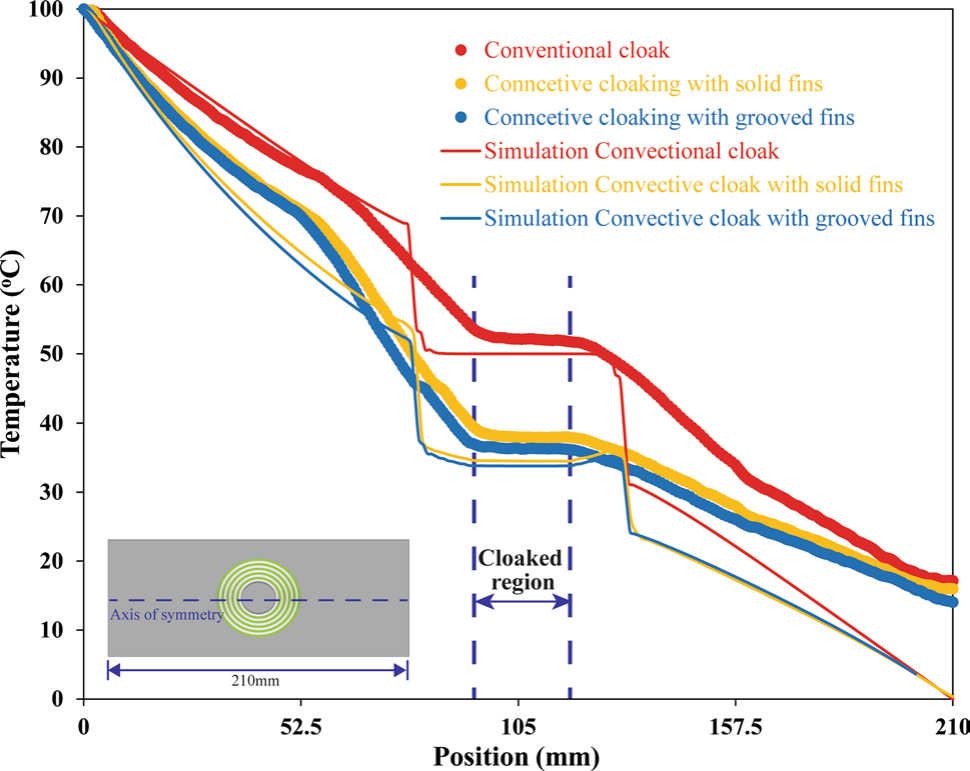
Comparison of temperature distribution obtained from finite element simulation (Fluent) and experiments with solid fins structure and grooved fins structure. (The simulation results are obtained from Ansys-Fluent module version 19.2—https://www.ansys.com/, the experimental results are obtained from FLIR tool version 5.13.18031.2002—https://www.flir.com.au/products/flir-tools/ and compiled in Adobe illustrator version 17.0.0—https://www.adobe.com/au/products/illustrator.html).

To quantify the exact value of temperature due to conventional cloak and convective element cloak, a graph is plotted as seen in Fig. 6. Figure 6 illustrates the temperature at the axis of symmetry of the specimen which covers all the regions e.g., baseplate, transformed thermal metamaterial and the cloaked region. At the baseplate region, the temperature gradient was uniform which indicates the uniform temperature distribution without any disturbance. Once it reached the thermal metamaterial region e.g., concentric rings, there was an abrupt change in the temperature due to the overall high anisotropic thermal conductivity in this region. Moreover, within the cloaked region, the temperature gradient became zero as if nothing was placed there. Once the cloaked region was over, thermal metamaterial region transformed the heat flux flow and returned to its path and the temperature gradient became constant again at the baseplate region towards the heat sink. The temperature at each point obtained by experiments was significantly aligned with fluent simulation results with a maximum deviation of ± 2 °C at the cloaked region. The temperature observed at the heat sink in the experimental analysis was approximately 20 °C in comparison with the simulation which was 0 °C. It indicated that the temperature contours were taken at the specimen end which was open to the environment. Due to the environment temperature (23 °C), the heat sink end of the specimen temperature was observed to be 20 °C. The temperature at the cloaked region measured as 52 °C for conventional cloaking, whereas convective element cloaking with attached fins reduced the cloak temperature to approximately 36 °C. The key reason for the reduction in temperature was the attached fin array that assisted the heat flow into the surrounding air via natural convection mechanism. Furthermore, since copper fins were used, heat transferred from the baseplate to the tip of the fin rapidly due to high thermal conductivity. In this study, two types of fin structures were considered in the experimental analysis, for instance, (i) solid fins and (ii) grooved fins. Experimental results confirmed that the grooved fins structure reduced the temperature further (by approximately 1 °C) than that of the solid fins structure as shown in Fig. 6. It was as expected due to enhance the airflow path in the grooved fins structure in comparison with solid fins structure.

### Numerical analysis for optimal fin array design parameters

**Figure 7:**
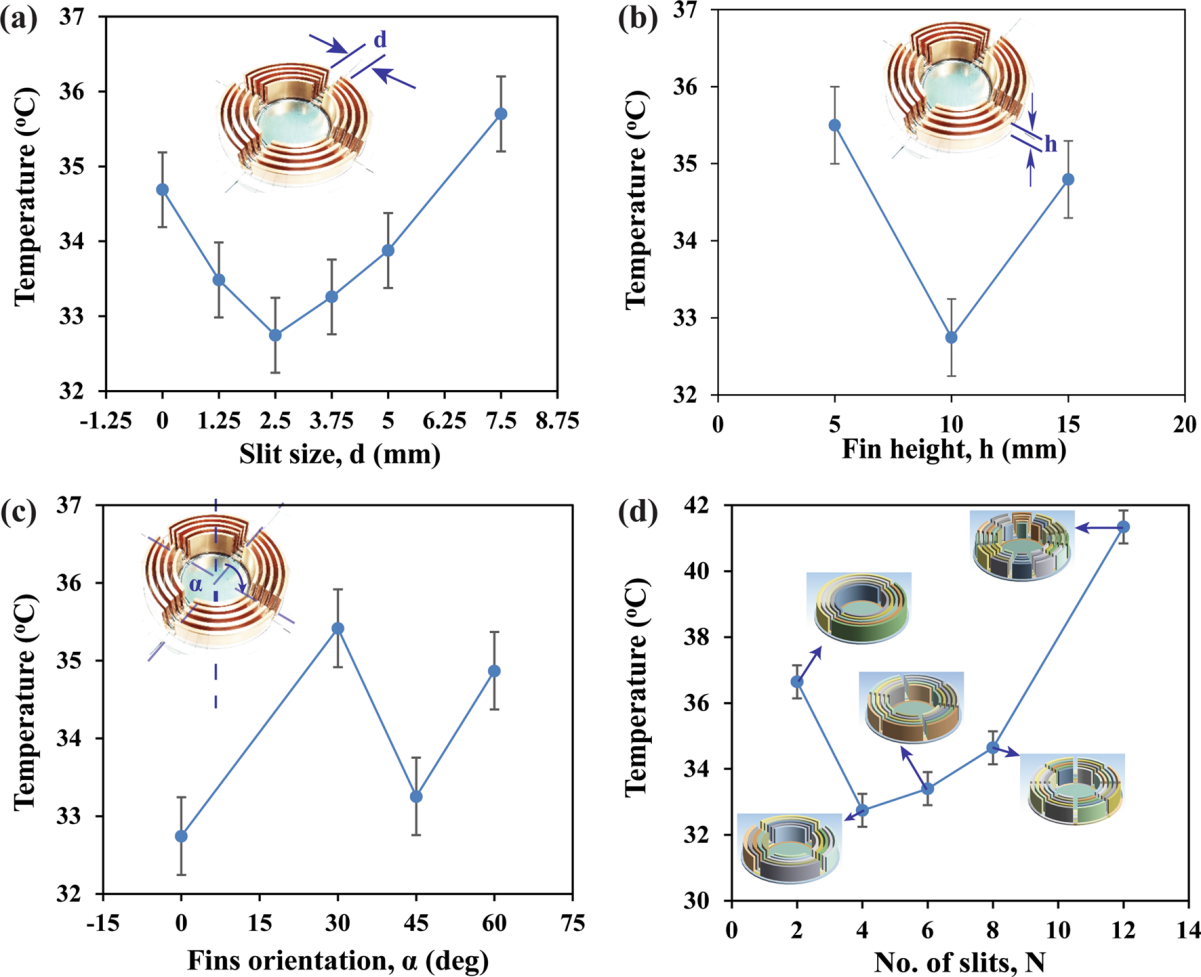
Characteristics fin array design analysis via Fluent CFD, Ansys. (**a**) slit gap, d, (**b**) fin height, h, (**c**) fin position, α, (**d**) number of slits per layer, N. (The results are obtained from Ansys-Fluent module version 19.2—https://www.ansys.com/ and compiled in Adobe illustrator version 17.0.0—https://www.adobe.com/au/products/illustrator.html).

To better understand the impact of airflow path on fin performance, a detailed investigation of fins characteristics under natural convection was carried out using Ansys-Fluent. Different sizes of fins were modelled by changing the slit size (d), fin height (h), fin orientation (α) and number of slits (N) and their results were presented in Fig. 7. Two main parameters that influence the efficiency of fins for convection heat transfer are smooth airflow path and effective surface area for heat convection^47^. In Fig. 7a, slit size represents the gap between fins where slit size, d = 0 implies the solid fins. Results of slit size (d) in Fig. 7a illustrated that the most effective slits/grooves gap should be 2.5 mm to achieve the lowest temperature within the cloaked region. In solid fins, effective surface area for heat convection is maximum. Whereas the airflow gets chocked due to limited space for airflow which reduced the overall convection heat transfer, hence increased cloaked temperature. Smooth airflow was significant in natural convection to achieve maximum efficiency because the driving force for air to flow was the buoyancy effect. When the slit size, (d > 2.5 mm), the airflow was smooth due to the large gap, but the effective surface area reduced significantly. In this manner, overall convection was reduced. The optimal slit gap achieved for this case is 2.5 mm at which the lowest temperature was achieved at the cloaked region due to the optimal trade-off between smooth airflow path and effective surface area^48^.

Three different fin heights (h) were considered and results were presented in Fig. 7b, where the fin thickness and fin gap were kept at 1 mm. Results showed that the fin height (h) influenced on the temperature at the cloaked region significantly. At fin height (h = 5 mm), due to the reduced surface area, heat transfer via convection becomes lower which increased the temperature. However, for fin height (h = 15 mm), the temperature at the cloaked region increased as well. It was due to the undesirable increase in height compared to specified fin spacing. At greater height (h = 15 mm) and compact fins array, the airflow chocked between the fins as the gap within the concentric fins was 1 mm. It should be avoided to increase fin height when fin spacing in fin array design is considerably small. Therefore, the optimal fin height (h) to achieve the minimum cloak temperature was 10 mm.

Figure 7c illustrates the cloak temperature with respect to the orientation of the concentric fins (α). The orientation was 0° when the slits were aligned with the axis of symmetry. As seen in Fig. 7c, the lowest temperature was achieved at the cloaked region when fins were aligned with the line of symmetry or rotated at 45°. The fin array was symmetrical at 45° as well which made the airflow uniform due to the buoyancy effect. However, at 30° or 60^o^ orientation, the asymmetric fin array increased the temperature and decreased the fin effectiveness. Therefore, it is anticipated that the fin array orientation (α) away from the symmetry will negatively influence on the convection heat transfer via fins. Finally, the influence of the number of slits (N) on the cloak temperature was investigated and their outcomes were presented in Fig. 7d. The results showed that the temperature dropped as the number of slits increased up to four. As for slit number (N = 2), the effective surface area was higher however the airflow gets constrained. On the other hand, when slit number (N) was greater than 4, air can easily flow within the fins for smooth natural convection due to more slits, but then the effective surface area reduced. Thus, the overall efficiency of fins for convection reduced, which increased the temperature of the cloaked region. The minimum cloak temperature was achieved as ~ 32.8 °C when the number of slits (N) is 4. From this investigation, it was clear that the cloaked region temperature was highly influenced by the fin array design. The optimal design for the concentric fins structure, for controlling the temperature within the cloaked region, should maintain the height (h = 10 mm), the number of slits (N = 4), with the slit gap (d) at 2.5 mm to attain the maximum convection heat transfer via fins.

## Conclusions

This study focused on the protection of heat-sensitive components by extending conventional thermal cloaking to convective element cloaking, combined heat conduction–convection transfer, via a passive cooling mechanism, surface fins. Both experiments and finite element simulations were conducted to examine the design. The concise summary of the obtained results are as follows:

(i) Temperature reduction around heat-sensitive components: The proposed convective element cloaking significantly reduced the temperature within the cloaked region, by ~ 15 °C, in comparison with convectional cloaking which was at ~ 50 °C, even if it is being used for an extended period of time, up to 8hours. Experimental analysis demonstrated fin array exhibit passive cooling, natural convection, around the cloaked region without disturbing the cloaking phenomenon.
(ii) Optimization of fin array design: Fluent simulations predicted an increase in fin effectiveness and heat dissipation via natural convection when optimal fin numbers and spacing between slits is considered. Increasing fin height (h) and number of fins per layer (N) negatively influence the fin effectiveness for a compact fin array design. Consequently, an optimal fin array design involves a trade-off between heat transfer surface area and the amount of heat transfer in natural convection.

This proposed design can keep the heat-sensitive components invisible to the heat flow and reduce the temperature around heat-sensitive components and hence can enhance the performance and longevity of electronic devices.

## Fabrication and experimental procedures

**Figure 8:**
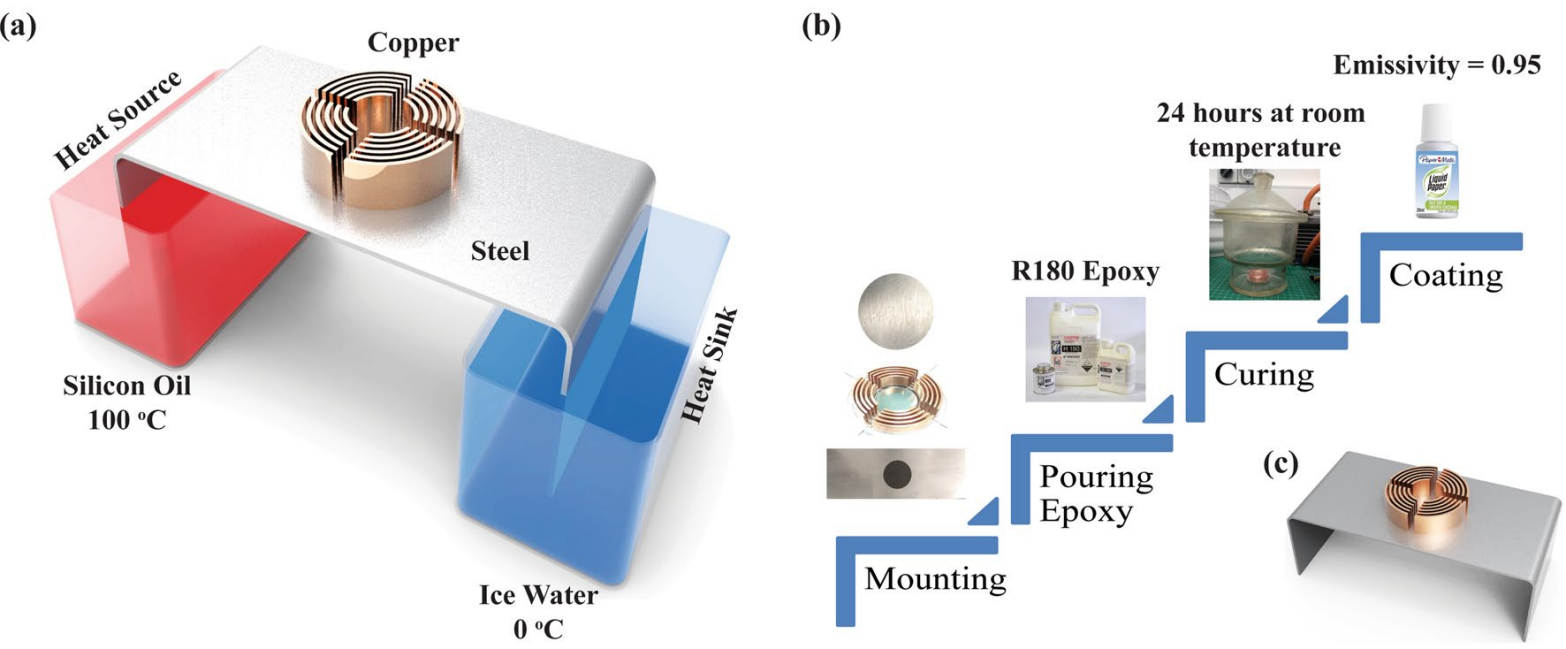
(**a**) Schematic of the experimental setup. (**b**) The step-by-step fabrication process of specimens. (**c**) Fabricated specimen to conduct experiments. (This figure is created in Adobe illustrator version 17.0.0—https://www.adobe.com/au/products/illustrator.html).

To validate these FEM simulation results, experiments were conducted at room temperature (23 °C). The detailed experimental setup is schematically shown in Fig. 8. Constant temperature heat source and heat sink were used to provide consistent temperature throughout the experiment. To maintain 100 °C at the heat source, polydimethylsiloxane (PDMS) based silicone oil was used as a heat source liquid in the water-bath due to its high boiling temperature (working range -50 to 200 °C). For heat sink, ice water bath was used to keep the temperature at 0 °C. The baseplate and the cloaked region material, as a reference, were steel (K_steel_ = 16.8 W/m K). Thermal metamaterial around the cloaked region was made of isometrics, homogenous and concentric alternating rings of epoxy (K_epoxy_ = 0.294 W/m K) and copper (K_copper_ = 400 W/m K), respectively. The concentric fins, made of copper, were attached to the copper layers as shown in Fig. 8b. The thickness of each ring, as well as fins, were kept at 1 mm.

Four specimens were fabricated to investigate the performance of convective cloaking for the temperature value within the cloaked region. The fabrication process is described in Fig. 8, which consists of four stages. In stage 1, all the components e.g., baseplate, concentric copper rings with attached fins and cloaked region was machined and aligned to make the specimen. In stage 2, liquid epoxy resin (R180) was poured to attach the thermal metamaterial layers of copper and epoxy with the base plate and cloaked region. In stage 3, the specimen was kept in a vacuum chamber at room temperature of ~ 23 °C for 24 h to remove air bubbles and to cure the epoxy resin. Since every material has a different emissivity, for example, baseplate and cloaked region (ε_steel_ = 0.55–0.61), fins (ε_copper_ = 0.07), concentric rings (ε_epoxy_ = 0.95)^49^. However, high emissivity coating (liquid paper corrector ε = 0.95)^50^ was used in stage 4 to attain an identical emissivity throughout the specimen. It aided to capture temperature contours using infrared thermal imaging camera FLIR E-40, which has accuracy of ± 2 °C and ranges up to 650 °C. Thermocouples, Delta OHM HD2328 (accuracy of ± 0.1 °C and ranges up to 600 °C) were also used to verify the temperature recorded by the infrared camera. Each experiment was conducted for 8hours and data was recorded every 15minutes and each experiment was repeated three times for its reliability.
